# Supplementary material for: Orthogonal regulation of phytochrome B abundance by stress-specific plastidial retrograde signaling metabolite
Source: Nat Commun. 2019 Jul 2;10:2904. doi: 10.1038/s41467-019-10867-w (PMC6606753; doi:10.1038/s41467-019-10867-w)
Supplement: Supplementary file 1 — Supplementary Information [file 41467_2019_10867_MOESM1_ESM.pdf]

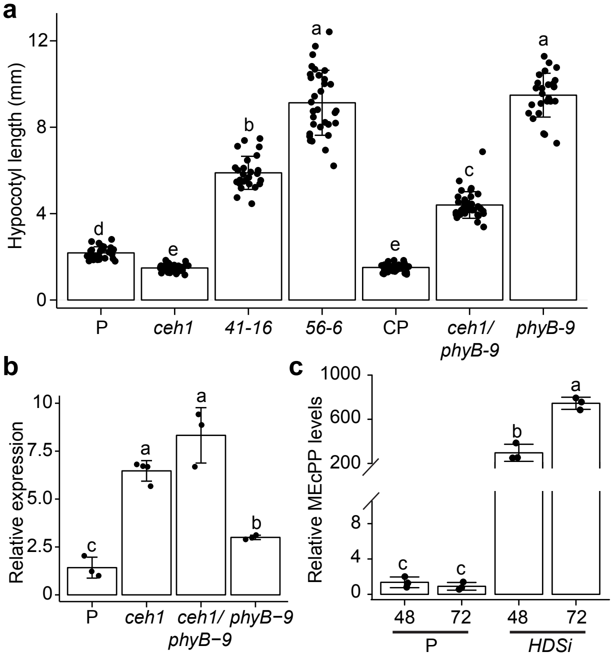

Supplementary Figure 1. PhyB is not a regulatory component of *HPL* expression

a Quantification of hypocotyl lengths of 7-day-old LD (16 h light/8 h dark) grown P, *ceh1*, *41-16*, *56-6*, complementation line (CP), *ceh1*/*phyB-9* and *phyB-9* seedlings. Data are mean ± SD, n ≥ 25. **b** Total RNAs isolated from 7-day-old seedlings of P, *ceh1*, *ceh1*/*phyB-9* and *phyB-9* were subjected to qRT-PCR analyses. The *HPL* transcript levels were normalized to the levels of At4g26410 (M3E9). Data are mean ± SD of three biological replicates and three technical replicates. **c** MEcPP levels of P and *HDSi* lines at 48 and 72 hours post DEX induction. The gap on the Y axis shows the scale change. Letters above bars indicate significant differences determined by Tukey's HSD method ($P < 0.05$).

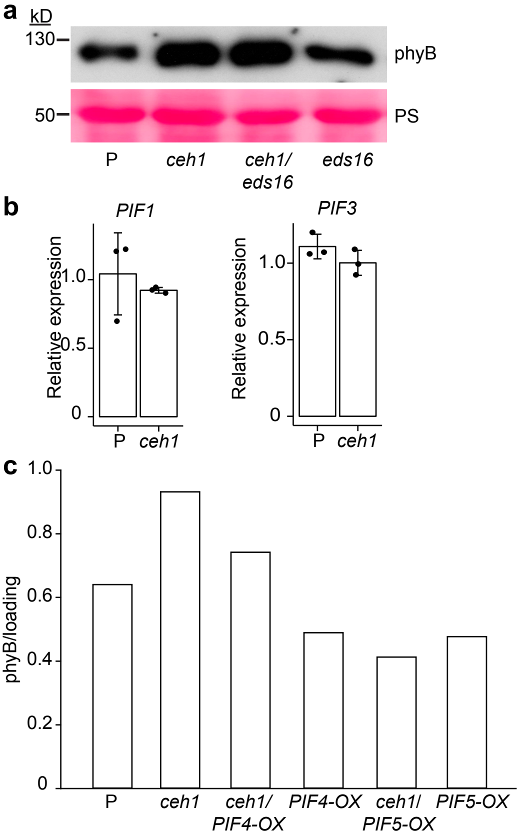

Supplementary Figure 2. PhyB abundance in *ceh1* is SA-independent

a phyB protein abundance in 7-day-old LD (16 h light/8 h dark) grown P, *ceh1*, *ceh1*/*eds16* and *eds16* seedlings. Ponceau S (PS) staining was used as the loading control. **b** *PIF1* and *PIF3* expression levels in P and *ceh1*. The experiment was performed on P and *ceh1* seedlings as described in Fig. S1**b**. Two-tailed Student's *t* tests display no significant differences of *PIF1* and *PIF3* expression levels between the two genotypes. **c** Quantification of phyB signal intensity of immunoblot analyses displayed in Fig. 4**e**.

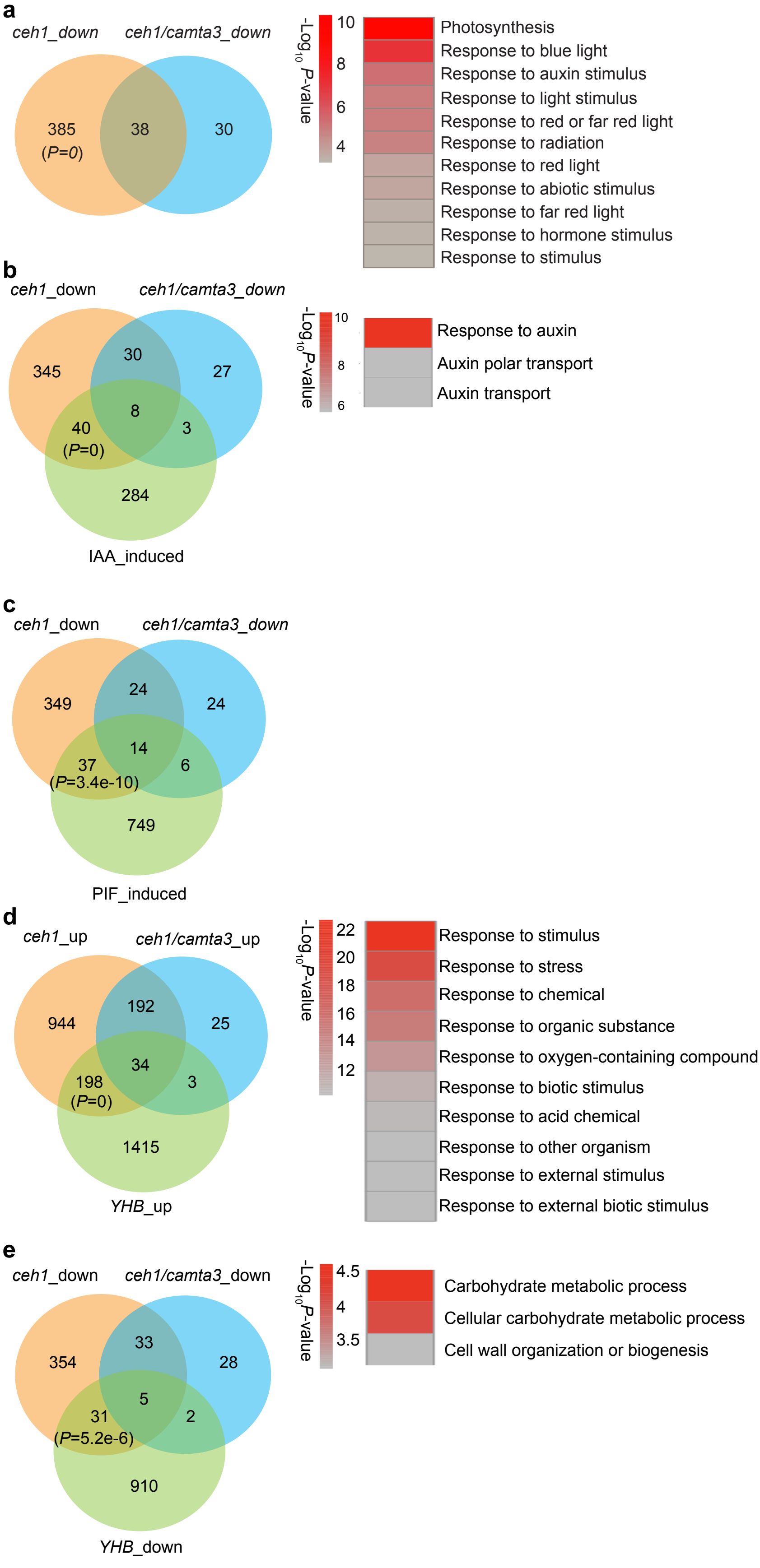

Supplementary Figure 3. Overlap between CAMTA3-, IAA-, PIF- and *YHB*-regulated genes

a Venn diagram shows that 91% of down-regulated genes in *ceh1* are CAMTA3 dependent. **b** Venn diagram shows that ~10.4% of CAMTA3-dependent down-regulated genes in *ceh1* are induced by IAA. **c** Venn diagram shows that ~9.6% of CAMTA3-dependent down-regulated genes in *ceh1* are induced in PIF. **d** Venn diagram shows that ~17.3% of CAMTA3-dependet up-regulated genes in *ceh1* are also up regulated in *YHB*. **e** Venn diagram shows that ~8.1% of CAMTA3-dependent down-regulated genes in *ceh1* are also down regulated in *YHB*. Heatmap of gene ontology (GO) enrichment analysis were shown next to the corresponding Venn diagram. Color intensity indicates *P*-value of each GO term.

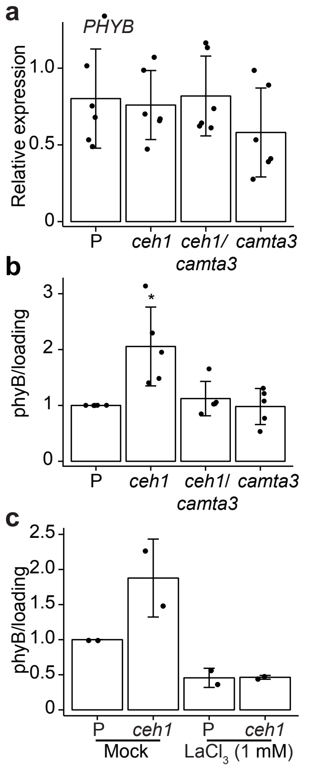

Supplementary Figure 4. PhyB protein abundance is CAMTA3-dependent

a *PHYB* expression levels in 7-day-old P, *ceh1*, *ceh1*/*camta3* and *camta3* seedlings show no significant differences in the transcript levels of these genotypes. Data are mean ± SD of six biological replicates and three technical replicates. **b** Quantification of phyB signal intensity of immunoblot analyses displayed in Fig. 7**b**. Data are mean ± SE, n=5. **c** Quantification of phyB immunoblot signal intensity of P and *ceh1* seedlings from Fig. 7**d**. Data are mean ± SE, n=2. Asterisks denote significant differences as determined by a two-tailed Student's *t* tests with a significance of $P < 0.05$.

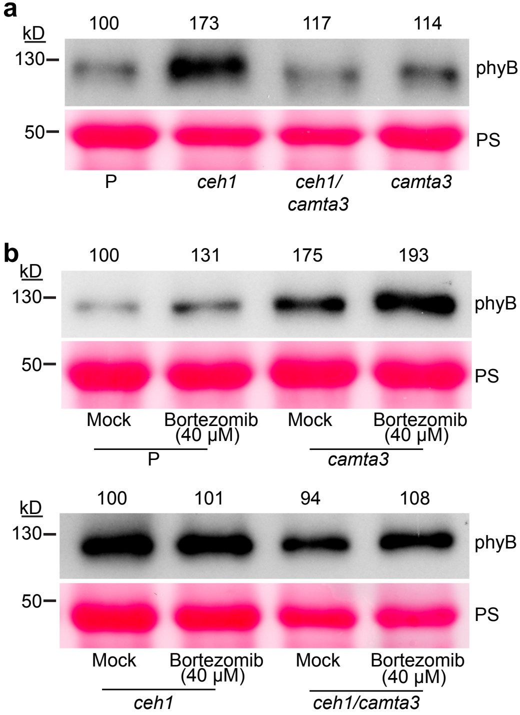

Supplementary Figure 5. CAMTA3 stabilizes phyB protein abundance

a phyB protein abundance in non-treated 7-day-old LD (16 h light/8 h dark) grown P, *ceh1*, *ceh1*/*camta3* and *camta3* seedlings. **b** phyB protein abundance in 7-day-old LD grown P, *ceh1*, *ceh1*/*camta3* and *camta3* seedlings in the absence (mock, 0.1% DMSO) and presence of proteasome inhibitor bortezomib. Ponceau S (PS) staining was used as loading control. Numbers on top of the immunoblot images represent normalized phyB abundance in various genotypes and in response to bortezomib treatment relative to the P (the two top panels) or *ceh1* (the bottom panel) seedlings.

Gene	Sequence	Note
PHYB-F	GCGATTGGTGGCCAAGAT	q-RT-PCR
PHYB-R	AAACTTCCCATTGCGGTCAA	q-RT-PCR
PIF1-F	ACTTATACCTCGCTGCAACAAGTC	q-RT-PCR
PIF1-R	CATGCCCGGATACATCATTGGC	q-RT-PCR
PIF3-F	GGGTTTGGGTTCAAAGAGAAG	q-RT-PCR
PIF3-R	TTGATCCTATCACGCCGTCTC	q-RT-PCR
PIF4-F	TCAGATGCAGCCGATGGAGATG	q-RT-PCR
PIF4-R	CGACGGTTGTTGACTTTGCTGTC	q-RT-PCR
PIF5-F	ACTCATACCTCACTGCAGCAGAAC	q-RT-PCR
PIF5-R	CCACTTCCCATCCACATCACTTGG	q-RT-PCR
HPL-F	GCTGAGAACGGTTGGAAAAC	q-RT-PCR
HPL-R	TCCGGCGATTAAGAGAGAAG	q-RT-PCR
AT4G26410-F	GAGCTGAAGTGGCTTCCATGAC	q-RT-PCR
AT4G26410-R	GGTCCGACATACCCATGATCC	q-RT-PCR
TIR1-F	TTTGGTACCTGACGGATGGG	Genotyping
TIR1-R	CGCAGCAAAACCTATATGCCC	Genotyping
HDS-F	AGGTGGTTCTCCCGGAAAAATCGAT	Genotyping
HDS-R	TCTCTCACAGTTTTCAAAGAATGG	Genotyping

Supplementary Table 1. List of primers used in PCR analyses
