# Supplementary material for: Orthogonal regulation of phytochrome B abundance by stress-specific plastidial retrograde signaling metabolite
Source: Nat Commun. 2019 Jul 2;10:2904. doi: 10.1038/s41467-019-10867-w (PMC6606753; doi:10.1038/s41467-019-10867-w)
Supplement: Supplementary file 3 — Description of Additional Supplementary Files [file 41467_2019_10867_MOESM3_ESM.pdf]

**Description of Additional Supplementary Files**

File Name: Supplementary Data 1
Description: Differentially expressed genes in ceh1 and ceh1/camta3

File Name: Supplementary Data 2
Description: List of CAMTA3-suppressed genes overlapped with IAA-induced genes

File Name: Supplementary Data 3
Description: List of CAMTA3-suppressed genes overlapped with PIF-induced genes

File Name: Supplementary Data 4
Description: List of CAMTA3-induced genes overlapped with YHB-induced genes

File Name: Supplementary Data 5
Description: List of CAMTA3-suppressed genes overlapped with YHB-suppressed genes
