# Supplementary material for: Orthogonal regulation of phytochrome B abundance by stress-specific plastidial retrograde signaling metabolite
Source: Nat Commun. 2019 Jul 2;10:2904. doi: 10.1038/s41467-019-10867-w (PMC6606753; doi:10.1038/s41467-019-10867-w)
Supplement: Supplementary file 5 — Supplementary Data 2 [file 41467_2019_10867_MOESM5_ESM.docx]

Supplementary Data 2. List of CAMTA3-suppressed genes overlapped with IAA-induced genes.

| **Gene ID** | **Gene Description** | **Gene Symbols** |
| --- | --- | --- |
| AT5G55250 | Encodes an enzyme which specifically converts IAA to its methyl ester form MelIAA. This gene belongs to the family of carboxyl methyltransferases whose members catalyze the transfer of the methyl group from S-adenosyl-L-methionine to carboxylic acid-containing substrates to form small molecule methyl esters. Expression of TCP genes is downregulated in mutant iamt1-D. | (AtIAMT1);IAA CARBOXYLMETHYLTRANSFERASE 1 (IAMT1) |
| AT3G06370 | member of Sodium proton exchanger family | (ATNHX4); SODIUM HYDROGEN EXCHANGER 4 (NHX4) |
| AT3G58120 | Encodes a member of the BZIP family of transcription factors. Forms heterodimers with the related protein AtbZIP34. Binds to G-boxes in vitro and is localized to the nucleus in onion epidermal cells. | (BZIP61); (ATBZIP61) |
| AT5G47370 | homeobox-leucine zipper genes induced by auxin, but not by other phytohormones. Plays opposite roles in the shoot and root tissues in regulating auxin-mediated morphogenesis. | (HAT2) |
| AT2G22810 | key regulatory enzyme in the biosynthesis of the plant hormone ethylene. ACS4 is specifically induced by indoleacetic acid (IAA). | 1-AMINOCYCLOPROPANE-1-CARBOXYLATE SYNTHASE 4 (ACS4);1-AMINOCYCLOPROPANE-1-CARBOXYLIC ACID SYNTHASE POLYPEPTIDE (ACC4); (ATACS4) |
| AT5G66080 | Protein phosphatase 2C family protein | ARABIDOPSIS PP2C CLADE D 9 (APD9) |
| AT4G14560 | auxin (indole-3-acetic acid) induced gene (IAA1) encoding a short-lived nuclear-localized transcriptional regulator protein. The mRNA is cell-to-cell mobile. | AUXIN RESISTANT 5 (AXR5);INDOLE-3-ACETIC ACID INDUCIBLE 1 (IAA1) |
| AT3G13980 | SKI/DACH domain protein | BIG GRAIN 4 (BG4) |
| AT1G69160 | suppressor | BIG GRAIN LIKE 1 (BGL1) |
| AT5G59010 | kinase with tetratricopeptide repeat domain-containing protein | BRASSINOSTEROID-SIGNALING KINASE 5 (BSK5) |
| AT1G64640 | early nodulin-like protein 8 | EARLY NODULIN-LIKE PROTEIN 8 (ENODL8); (AtENODL8) |
| AT5G64770 | Encodes a root meristem growth factor (RGF). Belongs to a family of functionally redundant homologous peptides that are secreted, tyrosine-sulfated, and expressed mainly in the stem cell area and the innermost layer of central columella cells. RGFs are required for maintenance of the root stem cell niche and transit amplifying cell proliferation. Members of this family include: At5g60810 (RGF1), At1g13620 (RGF2), At2g04025 (RGF3), At3g30350 (RGF4), At5g51451 (RGF5), At4g16515 (RGF6), At3g02240 (RGF7), At2g03830 (RGF8) and At5g64770 (RGF9). | GOLVEN 2 (GLV2); CLE-LIKE 9 (CLEL 9); ROOT MERISTEM GROWTH FACTOR 9 (RGF9) |
| AT1G04240 | SHY2/IAA3 regulates multiple auxin responses in roots. It is induced rapidly by IAA and has been shown to be phosphorylated by oat phytochrome A in vitro. | INDOLE-3-ACETIC ACID INDUCIBLE 3 (IAA3); SHORT HYPOCOTYL 2 (SHY2) |
| AT5G43700 | Auxin inducible protein similar to transcription factors. | INDOLE-3-ACETIC ACID INDUCIBLE 4 (IAA4); AUXIN INDUCIBLE 2-11 (ATAUX2-11) |
| AT4G17460 | Encodes a class II HD-ZIP protein that regulates meristematic activity in different tissues, and that it is necessary for the correct formation of the gynoecium. | JAIBA (JAB); (HAT1) |
| AT3G29370 | Encodes an atypical member of the bHLH (basic helix-loop-helix) family transcriptional factors. | P1R3 (P1R3) |
| AT5G04190 | Encodes phytochrome kinase substrate 4, a phytochrome signaling component involved in phototropism. It is phosphorylated in a phot1-dependent manner in vitro. Phosphorylation is transient and regulated by a type 2- protein phosphatase. | PHYTOCHROME KINASE SUBSTRATE 4 (PKS4) |
| AT1G23080 | Encodes a novel component of auxin efflux that is located apically in the basal cell and is involved during embryogenesis in setting up the apical-basal axis in the embryo. It is also involved in pattern specification during root development. In roots, it is expressed at lateral and basal membranes of perivascular cells in the meristem and elongation zone, whereas in the columella cells it coincides with the PIN3 domain. Plasma membrane-localized PIN proteins mediate a saturable efflux of auxin. PINs mediate auxin efflux from mammalian and yeast cells without needing additional plant-specific factors. The action of PINs in auxin efflux is distinct from PGPs, rate-limiting, specific to auxins and sensitive to auxin transport inhibitors. PINs are directly involved of in catalyzing cellular auxin efflux. | PIN-FORMED 7 (PIN7); ARABIDOPSIS PIN-FORMED 7 (ATPIN7) |
| AT3G03840 | SAUR-like auxin-responsive protein family | SMALL AUXIN UP RNA 27 (SAUR27) |
| AT1G29440 | SAUR-like auxin-responsive protein family | SMALL AUXIN UP RNA 63 (SAUR63) |
| AT1G29490 | SAUR-like auxin-responsive protein family | SMALL AUXIN UPREGULATED 68 (SAUR68) |
| AT4G34760 | SAUR-like auxin-responsive protein family | SMALL AUXIN UPREGULATED RNA 50 (SAUR50) |
| AT1G29430 | SAUR762 expression is induced during pollination and expressed in pollen tubes. SAUR62 likely functions in translation of proteins required for pollen tube development/function. | SMALL AUXIN UPREGULATED RNA 62 (SAUR62) |
| AT1G29460 | SAUR-like auxin-responsive protein family | SMALL AUXIN UPREGULATED RNA 65 (SAUR65) |
| AT1G29510 | This locus was referred to as SAUR68 in PMID:17948056 but the nomenclature should be SAUR67. | SMALL AUXIN UPREGULATED RNA 67 (SAUR67); SMALL AUXIN UPREGULATED 68 (SAUR68) |
| AT5G07000 | Encodes a member of the sulfotransferase family of proteins. Although it has 85% amino acid identity with ST2A (At5g07010), this protein is not able to transfer a sulfate group to 11- or 12-hydroxyjasmonic acid in vitro. It may be able to act on structurally related jasmonates. | SULFOTRANSFERASE 2B (ST2B); ARABIDOPSIS THALIANA SULFOTRANSFERASE 2B (ATST2B) |
| AT3G25710 | Encodes a basic helix-loop-helix transcription factor that is expressed in the hypophysis-adjacent embryo cells and is required and partially sufficient for MP-dependent root initiation. Involved in response to phosphate starvation. Negative regulator of root hair development, anthocyanin formation and Pi content. Its expression is responsive to both phosphate (Pi) and phosphite (Phi) in shoots. | TARGET OF MONOPTEROS 5 (TMO5); BASIC HELIX-LOOP-HELIX 32 (BHLH32); (ATAIG1) |
| AT1G68810 | basic helix-loop-helix (bHLH) DNA-binding superfamily protein | TMO5-LIKE1 (T5L1); ABNORMAL SHOOT 5 (ABS5) |
| AT1G65310 | Encodes a xyloglucan endotransglucosylase/hydrolase with only only the endotransglucosylase (XET; EC 2.4.1.207) activity towards xyloglucan and non-detectable endohydrolytic (XEH; EC 3.2.1.151) activity. Expressed in the mature or basal regions of both the main and lateral roots, but not in the tip of these roots where cell division occurs. | XYLOGLUCAN ENDOTRANSGLUCOSYLASE/HYDROLASE 17 (XTH17); XYLOGLUCAN ENDOTRANSGLUCOSYLASE/HYDROLASE 17 (ATXTH17) |
| AT1G11545 | xyloglucan endotransglucosylase/hydrolase 8 | XYLOGLUCAN ENDOTRANSGLUCOSYLASE/HYDROLASE 8 (XTH8) |
| AT2G32560 | F-box family protein |  |
| AT3G13000 | ubiquinone biosynthesis protein (Protein of unknown function, DUF547) |  |
| AT3G42800 | AF-like protein |  |
| AT4G09890 | mediator of RNA polymerase II transcription subunit, putative (DUF3511) |  |
| AT4G10150 | RING/U-box superfamily protein |  |
| AT4G18970 | GDSL-motif esterase/acyltransferase/lipase. Enzyme group with broad substrate specificity that may catalyze acyltransfer or hydrolase reactions with lipid and non-lipid substrates. |  |
| AT4G38820 | hypothetical protein |  |
| AT5G22860 | Serine carboxypeptidase S28 family protein |  |
| AT5G47800 | Phototropic-responsive NPH3 family protein |  |
| AT5G48900 | Pectin lyase-like superfamily protein |  |
