# Supplementary material for: Orthogonal regulation of phytochrome B abundance by stress-specific plastidial retrograde signaling metabolite
Source: Nat Commun. 2019 Jul 2;10:2904. doi: 10.1038/s41467-019-10867-w (PMC6606753; doi:10.1038/s41467-019-10867-w)
Supplement: Supplementary file 6 — Supplementary Data 3 [file 41467_2019_10867_MOESM6_ESM.docx]

Supplementary Data 3. List of CAMTA3-suppressed genes overlapped with PIF-induced genes

| **Gene ID** | **Gene Description** | **Gene Symbols** |
| --- | --- | --- |
| AT1G04240 | SHY2/IAA3 regulates multiple auxin responses in roots. It is induced rapidly by IAA and has been shown to be phosphorylated by oat phytochrome A in vitro. | SHORT HYPOCOTYL 2 (SHY2) |
| AT1G06980 | 6,7-dimethyl-8-ribityllumazine synthase |  |
| AT1G19510 | RAD-like 5 | RAD-LIKE 5 (RL5) |
| AT1G22740 | GTP-binding protein Rab7 | RAB GTPASE HOMOLOG G3B (RABG3B) |
| AT1G26290 | hypothetical protein |  |
| AT1G26945 | Encodes a basic helix-loop-helix (bHLH) protein involved in blue/far-red light signaling. Physically interacts with HFR1 and negatively regulates its activity. | KIDARI (KDR) |
| AT1G64640 | early nodulin-like protein 8 | EARLY NODULIN-LIKE PROTEIN 8 (ENODL8) |
| AT1G67265 | ROTUNDIFOLIA like 21 | ROTUNDIFOLIA LIKE 21 (RTFL21) |
| AT1G73830 | Encodes the brassinosteroid signaling component BEE3 (BR-ENHANCED EXPRESSION 3). Positively modulates the shade avoidance syndrome in Arabidopsis seedlings. | BR ENHANCED EXPRESSION 3 (BEE3) |
| AT1G78230 | Outer arm dynein light chain 1 protein |  |
| AT2G11810 | MGD3 is the major enzyme for galactolipid metabolism during phosphate starvation. Does not contribute to galactolipid synthesis under P1-sufficient conditions. | MONOGALACTOSYLDIACYLGLYCEROL SYNTHASE TYPE C (MGDC) |
| AT2G26695 | Ran BP2/NZF zinc finger-like superfamily protein |  |
| AT2G38980 | pseudogene of cationic amino acid transporter 4 |  |
| AT2G42870 | Encodes PHYTOCHROME RAPIDLY REGULATED1 (PAR1), an atypical basic helix-loop-helix (bHLP) protein. Closely related to PAR2 (At3g58850). Up regulated after simulated shade perception. Acts in the nucleus to control plant development and as a negative regulator of shade avoidance response. Functions as transcriptional repressor of auxin-responsive genes SAUR15 (AT4G38850) and SAUR68 (AT1G29510). | PHY RAPIDLY REGULATED 1 (PAR1) |
| AT3G04700 | carboxylate clamp-TPR protein (DUF1685) |  |
| AT3G05900 | neurofilament protein-like protein |  |
| AT3G07380 | glycosyltransferase family protein (DUF23) |  |
| AT3G10550 | Has 3'-phosphatase activity against both phosphatidylinositol-3,5-bisphosphate (PtdIns3,5P2) and Phosphatidylinositol-3-phosphate (PtdIns3P). The in vitro activity was higher with PtdIns3,5P2 than with PtdIns3P. | MYOTUBULARIN 1 (MTM1) |
| AT3G16120 | Dynein light chain type 1 family protein |  |
| AT3G21330 | basic helix-loop-helix (bHLH) DNA-binding superfamily protein |  |
| AT3G26510 | Octicosapeptide/Phox/Bem1p family protein |  |
| AT3G30180 | Encodes a cytochrome p450 enzyme that catalyzes the last reaction in the production of brassinolide. It is capable of converting 6-deoxocastasterone into castasterone, a C-6 oxidation, as well as the further conversion of castasterone into brassinolide by a Baeyer-Villinger oxidation reaction at C-6, resulting in the formation of an unusual seven-membered lactone ring. The enzyme possesses high affinity for both C28- and C27-Brassinosteroids. The expression of the gene using a CYP85A2 promoter:LUC fusion construct was shown to be under circadian and light control. | BRASSINOSTEROID-6-OXIDASE 2 (BR6OX2) |
| AT3G54830 | Transmembrane amino acid transporter family protein |  |
| AT3G58850 | Encodes PHYTOCHROME RAPIDLY REGULATED2 (PAR2), an atypical basic helix-loop-helix (bHLP) protein. Closely related to PAR1 (At2g42870). Up regulated after simulated shade perception. Acts in the nucleus to control plant development and as a negative regulator of shade avoidance response. Functions as transcriptional repressor of auxin-responsive genes SAUR15 (AT4G38850) and SAUR68 (AT1G29510). | PHY RAPIDLY REGULATED 2 (PAR2) |
| AT4G01335 | TATA box-binding protein associated factor RNA polymerase I subunit B-like protein |  |
| AT5G19600 | Encodes sulfate transporter Sultr3;5. | SULFATE TRANSPORTER 3;5 (SULTR3;5) |
| AT5G23750 | Remorin family protein |  |
| AT5G24580 | Heavy metal transport/detoxification superfamily protein |  |
| AT5G35525 | PLAC8 family protein |  |
| AT5G41080 | Encodes a member of the glycerophosphodiester phosphodiesterase (GDPD) family. | GLYCEROPHOSPHODIESTER PHOSPHODIESTERASE 2 (GDPD2) |
| AT5G43290 | member of WRKY Transcription Factor; Group II-c | WRKY DNA-BINDING PROTEIN 49 (WRKY49) |
| AT5G46240 | Encodes a potassium channel protein (KAT1). ABA triggers KAT1 endocytosis both in epidermal cells as well as guard cells. Upon removal of ABA, KAT1 is recycled back to the plasma membrane. KAT1 is localized within 0.5?0.6 μm diameter microdomains at the plasma membrane surface. KAT1 belongs to the Shaker family K+ channel. This family includes five groups based on phylogenetic analysis (FEBS Letters (2007) 581: 2357): I (inward rectifying channel): AKT1 (AT2G26650), AKT5 (AT4G32500) and SPIK (also known as AKT6, AT2G25600); II (inward rectifying channel): KAT1 (AT5G46240) and KAT2 (AT4G18290); III (weakly inward rectifying channel): AKT2 (AT4G22200); IV (regulatory subunit involved in inwardly rectifying conductance formation): KAT3 (also known as AtKC1, AT4G32650); V (outward rectifying channel): SKOR (AT3G02850) and GORK (AT5G37500). | POTASSIUM CHANNEL IN ARABIDOPSIS THALIANA 1 (KAT1) |
| AT5G48900 | Pectin lyase-like superfamily protein |  |
| AT5G52280 | Myosin heavy chain-related protein |  |
| AT5G59010 | kinase with tetratricopeptide repeat domain-containing protein | BRASSINOSTEROID-SIGNALING KINASE 5 (BSK5) |
| AT5G66080 | Protein phosphatase 2C family protein | ARABIDOPSIS PP2C CLADE D 9 (APD9) |
