# Supplementary material for: Orthogonal regulation of phytochrome B abundance by stress-specific plastidial retrograde signaling metabolite
Source: Nat Commun. 2019 Jul 2;10:2904. doi: 10.1038/s41467-019-10867-w (PMC6606753; doi:10.1038/s41467-019-10867-w)
Supplement: Supplementary file 7 — Supplementary Data 4 [file 41467_2019_10867_MOESM7_ESM.docx]

Supplementary Data 4. List of CAMTA3-induced genes overlapped with *YHB*-induced genes

| **Gene ID** | **Gene Description** | **Gene Symbols** |
| --- | --- | --- |
| AT5G48900 | Pectin lyase-like superfamily protein |  |
| AT1G72230 | Cupredoxin superfamily protein |  |
| AT1G15260 | LOW protein: ATP-dependent RNA helicase-like protein |  |
| AT4G18970 | GDSL-motif esterase/acyltransferase/lipase. Enzyme group with broad substrate specificity that may catalyze acyltransfer or hydrolase reactions with lipid and non-lipid substrates. |  |
| AT1G56720 | Protein kinase superfamily protein |  |
| AT5G61660 | glycine-rich protein |  |
| AT5G16030 | mental retardation GTPase activating protein |  |
| AT5G03760 | encodes a beta-mannan synthase that is required for agrobacterium-mediated plant genetic transformation involves a complex interaction between the bacterium and the host plant. 3' UTR is involved in transcriptional regulation and the gene is expressed in the elongation zone of the root. | (ATCSLA09); (CSLA09); RESISTANT TO AGROBACTERIUM TRANSFORMATION 4 (RAT4); CELLULOSE SYNTHASE LIKE A9 (CSLA9); (ATCSLA9) |
| AT3G58120 | Encodes a member of the BZIP family of transcription factors. Forms heterodimers with the related protein AtbZIP34. Binds to G-boxes in vitro and is localized to the nucleus in onion epidermal cells. | (BZIP61); (ATBZIP61) |
| AT2G34620 | Mitochondrial transcription termination factor family member. | (MTERF10) |
| AT5G06530 | Encodes ABCG22, an ABC transporter gene. Mutation results in increased water transpiration and drought susceptibility. | ARABIDOPSIS THALIANA ATP-BINDING CASSETTE G22 (AtABCG22); ATP-BINDING CASSETTE G22 (ABCG22) |
| AT3G25710 | Encodes a basic helix-loop-helix transcription factor that is expressed in the hypophysis-adjacent embryo cells and is required and partially sufficient for MP-dependent root initiation. Involved in response to phosphate starvation. Negative regulator of root hair development, anthocyanin formation and Pi content. Its expression is responsive to both phosphate (Pi) and phosphite (Phi) in shoots. | TARGET OF MONOPTEROS 5 (TMO5); BASIC HELIX-LOOP-HELIX 32 (BHLH32); (ATAIG1) |
| AT2G28470 | putative beta-galactosidase (BGAL8 gene) | BETA-GALACTOSIDASE 8 (BGAL8) |
| AT3G13980 | SKI/DACH domain protein | BIG GRAIN 4 (BG4) |
| AT3G11550 | Uncharacterized protein family (UPF0497) | CASPARIAN STRIP MEMBRANE DOMAIN PROTEIN 2 (CASP2) |
| AT1G29395 | Integral membrane protein in the inner envelope of chloroplasts. Provide freezing tolerance. Expression is induced by short-term cold-treatment, water deprivation, and abscisic acid treatment. | COLD REGULATED 414 THYLAKOID MEMBRANE 1 (COR414-TM1); COLD REGULATED 314 THYLAKOID MEMBRANE 1 (COR413-TM1); COLD REGULATED 314 INNER MEMBRANE 1 (COR413IM1) |
| AT1G05470 | Encodes an inositol polyphosphate 5' phosphatase (5PTase) that is required for the proper recruitment of cells into developing vascular tissue in leaves and cotyledons. It is most similar to Type I 5PTases that are known to cleave a phosphate from IP3 or IP4. cvp2 mutants have elevated levels of IP3 and are hypersensitive to ABA in seed germination assays. | COTYLEDON VASCULAR PATTERN 2 (CVP2) |
| AT3G48720 | Encodes a hydroxycinnamoyl-CoA: v-hydroxy fatty acid transferase involved in cutin synthesis. Mutants are almost devoid of ferulic acid. | DEFICIENT IN CUTIN FERULATE (DCF) |
| AT1G64640 | early nodulin-like protein 8 | EARLY NODULIN-LIKE PROTEIN 8 (ENODL8); (AtENODL8) |
| AT5G66460 | Encodes an endo-beta-mannanase involved in seed germination and silique dehiscence. | (AtMAN7); ENDO-BETA-MANNASE 7 (MAN7) |
| AT2G21050 | Encodes LAX2 (LIKE AUXIN RESISTANT), a member of the AUX1 LAX family of auxin influx carriers. Required for the establishment of embryonic root cell organization. | LIKE AUXIN RESISTANT 2 (LAX2) |
| AT1G52190 | Encodes a low affinity nitrate transporter that is expressed in the plasma membrane and found in the phloem of the major veins of leaves. It is responsible for nitrate redistribution to young leaves. | NITRATE TRANSPORTER 1.11 (NRT1.11); (ATNPF1.2); NRT1/ PTR FAMILY 1.2 (NPF1.2) |
| AT2G22170 | Lipase/lipooxygenase, PLAT/LH2 family protein | PLAT DOMAIN PROTEIN 2 (PLAT2) |
| AT1G03630 | Encodes for a protein with protochlorophyllide oxidoreductase activity. The enzyme is NADPH- and light-dependent. | PROTOCHLOROPHYLLIDE OXIDOREDUCTASE C (POR C); (PORC) |
| AT3G06370 | member of Sodium proton exchanger family | (ATNHX4); SODIUM HYDROGEN EXCHANGER 4 (NHX4) |
| AT4G12970 | Encodes a cysteine-rich peptide, a secretory factor that is produced in the mesophyll cells and acts on the epidermis to increase stomatal formation. Its mature form is a 45-aa peptide with three intramolecular disulfide bonds. It is proposed that STOMAGEN increases stomatal number by competing with two negative regulators of stomatal density, EPF1 and EPF2. STOMAGEN has been shown to compete with EPF2 for binding to the ER and TMM receptor kinases.Binding of STOMAGEN to ER prevents induction of the EPF2-ER MAPK cascade. It's transcript levels change after inducing MUTE expression in a mute background. | STOMAGEN (STOMAGEN); EPIDERMAL PATTERNING FACTOR LIKE-9 (EPFL9); (ATEPFL9) |
| AT5G07000 | Encodes a member of the sulfotransferase family of proteins. Although it has 85% amino acid identity with ST2A (At5g07010), this protein is not able to transfer a sulfate group to 11- or 12-hydroxyjasmonic acid in vitro. It may be able to act on structurally related jasmonates. | SULFOTRANSFERASE 2B (ST2B); ARABIDOPSIS THALIANA SULFOTRANSFERASE 2B (ATST2B) |
| AT4G17340 | tonoplast intrinsic protein 2 | (DELTA-TIP2); TONOPLAST INTRINSIC PROTEIN 2;2 (TIP2;2) |
| AT2G22190 | Haloacid dehalogenase-like hydrolase (HAD) superfamily protein | TREHALOSE-6-PHOSPHATE PHOSPHATASE E (TPPE) |
| AT5G13870 | EXGT-A4, endoxyloglucan transferase, | XYLOGLUCAN ENDOTRANSGLUCOSYLASE/HYDROLASE 5 (XTH5); ENDOXYLOGLUCAN TRANSFERASE A4 (EXGT-A4) |
| AT1G11545 | xyloglucan endotransglucosylase/hydrolase 8 | XYLOGLUCAN ENDOTRANSGLUCOSYLASE/HYDROLASE 8 (XTH8) |
