# Supplementary material for: Orthogonal regulation of phytochrome B abundance by stress-specific plastidial retrograde signaling metabolite
Source: Nat Commun. 2019 Jul 2;10:2904. doi: 10.1038/s41467-019-10867-w (PMC6606753; doi:10.1038/s41467-019-10867-w)
Supplement: Supplementary file 8 — Supplementary Data 5 [file 41467_2019_10867_MOESM8_ESM.docx]

Supplementary Data 5. List of CAMTA3-suppressed genes overlapped with *YHB*-suppressed genes

| **Gene ID** | **Gene Description** | **Gene Symbols** |
| --- | --- | --- |
| AT1G49050 | Encodes a member of the aspartyl protease family. Interacts with BAGP1 and BAG6 and appears to be required for cleavage of BAG6 as BAG6 is not cleaved in APCB1 mutant backgrounds. | (APCB1) |
| AT1G67970 | member of Heat Stress Transcription Factor (Hsf) family | (AT-HSFA8); (ATHSFA8); HEAT SHOCK TRANSCRIPTION FACTOR A8 (HSFA8) |
| AT4G28390 | Encodes a mitochondrial ADP/ATP carrier protein. Shown in heterologous systems to be located in the plasma membrane. Has comparable affinity for ADP and ATP (in E.coli). | (ATAAC3); ADP/ATP CARRIER 3 (AAC3) |
| AT1G01720 | Belongs to a large family of putative transcriptional activators with NAC domain. Transcript level increases in response to wounding and abscisic acid. ATAF1 attenuates ABA signaling and synthesis. Mutants are hyposensitive to ABA. The mRNA is cell-to-cell mobile. | (ATAF1); (ANAC2); ARABIDOPSIS NAC DOMAIN CONTAINING PROTEIN 2 (ANAC002) |
| AT4G20860 | involved in the generation of H2O2 from reduced compounds | (ATBBE22); CELLODEXTRIN OXIDASE (CELLOX); BERBERINE BRIDGE ENZYME 22 (BBE22) |
| AT3G51860 | cation exchanger 3 | (ATHCX1); (CAX1-LIKE); (ATCAX3); CATION EXCHANGER 3 (CAX3) |
| AT2G41260 | Late-embryogenesis-abundant gene. Involved in the acquisition of desiccation tolerance during late phase of embryogenesis. | (ATM17); (M17) |
| AT5G64120 | Encodes a cell wall bound peroxidase that is induced by hypo-osmolarity and is involved in the lignification of cell walls. | (ATPRX71); PEROXIDASE 71 (PRX71) |
| AT5G10380 | Encodes a RING finger domain protein with E3 ligase activity that is localized to the lipid rafts of the plasma membrane. Expression is increased in response to fungal pathogen. May be involved in regulation of programmed cell death by facilitating degradation of regulation of PDC activators. The mRNA is cell-to-cell mobile. | (ATRING1); (RING1) |
| AT1G18480 | Calcineurin-like metallo-phosphoesterase superfamily protein | (ATSLP2); SHEWENELLA-LIKE PROTEIN PHOSPHATASE 2 (SLP2) |
| AT2G30250 | member of WRKY Transcription Factor; Group I. Located in nucleus. Involved in response to various abiotic stresses - especially salt stress. | (AtWRKY25); WRKY DNA-BINDING PROTEIN 25 (WRKY25) |
| AT4G12490 | Encodes a member of the AZI family of lipid transfer proteins. Contains a PRR domain that appears to be required for localization to the chloroplast. | (AZI3) |
| AT5G04340 | Encodes a C2H2 zinc finger transcription factor that coordinately activates phytochelatin-synthesis related gene expression and directly targets GSH1 by binding to its promoter to positively regulate Cd accumulation and tolerance. | (C2H2); (ATZAT6); ZINC FINGER OF ARABIDOPSIS THALIANA 6 (ZAT6); COLD INDUCED ZINC FINGER PROTEIN 2 (CZF2) |
| AT2G04030 | Encodes a chloroplast-targeted 90-kDa heat shock protein located in the stroma involved in red-light mediated deetiolation response and crucial for protein import into the chloroplast stroma. Mutants are resistant to chlorate, have elongated hypocotyls in light, and affect the expression of NR2, CAB, and RBCS but NOT NR1 and NiR. | (CR88); EMBRYO DEFECTIVE 1956 (emb1956); (ATHSP90C); HEAT SHOCK PROTEIN 88.1 (Hsp88.1); HEAT SHOCK PROTEIN 90.5 (AtHsp90.5); HEAT SHOCK PROTEIN 90.5 (HSP90.5) |
| AT3G22640 | cupin family protein | (PAP85) |
| AT2G31890 | Protein contains putative RNA binding domain. Expressed in response to Pseudomonas syringae infection. Resistance requires silencing of AtRAP suggesting it functions as a negative regulator of plant disease resistance. Alpha helical repeat protein; only member of the OPR (octotricopeptide repeat) protein family in land plants. | (RAP); (ATRAP) |
| AT4G15690 | Encodes a member of the CC-type glutaredoxin (ROXY) family that has been shown to interact with the transcription factor TGA2 and suppress ORA59 promoter activity. | (ROXY12); (GRXS5) |
| AT2G37970 | SOUL heme-binding family protein | (SOUL-1); (ATHBP2); HAEM-BINDING PROTEIN 2 (HBP2) |
| AT1G70700 | JAZ9 is a protein presumed to be involved in jasmonate signaling. JAZ9 transcript levels rise in response to a jasmonate stimulus. JAZ9 can interact with the COI1 F-box subunit of an SCF E3 ubiquitin ligase in a yeast-two-hybrid assay only in the presence of jasmonate-isoleucine (JA-ILE) or coronatine. The Jas domain appears to be important for JAZ9-COI1 interactions in the presence of coronatine. Two positive residues (R205 and R206) in the Jas domain shown to be important for coronatine -dependent COI1 binding are not required for binding AtMYC2. The mRNA is cell-to-cell mobile. | (TIFY7); JASMONATE-ZIM-DOMAIN PROTEIN 9 (JAZ9) |
| AT4G15490 | Encodes a protein that might have sinapic acid:UDP-glucose glucosyltransferase activity. | (UGT84A3) |
| AT1G36060 | encodes a member of the DREB subfamily A-6 of ERF/AP2 transcription factor family. The protein contains one AP2 domain. There are 8 members in this subfamily including RAP2.4.Overexpression results in increased drought tolerance and vitrified leaves. Binds to DRE/GCC promoter elements and activates expression of aquaporin genes AtTIP1;1, AtTIP2;3, and AtPIP2;2. | (WIND3); TRANSLUCENT GREEN (TG) |
| AT1G27730 | Related to Cys2/His2-type zinc-finger proteins found in higher plants. Compensated for a subset of calcineurin deficiency in yeast. Salt tolerance produced by ZAT10 appeared to be partially dependent on ENA1/PMR2, a P-type ATPase required for Li+ and Na+ efflux in yeast. The protein is localized to the nucleus, acts as a transcriptional repressor and is responsive to chitin oligomers. Also involved in response to photooxidative stress. | (ZAT10); SALT TOLERANCE ZINC FINGER (STZ) |
| AT2G01520 | Encodes a cis-cinnamic acid responsive gene that is a member of the major latex protein-like gene family and plays a role in promoting vegetative growth and delaying flowering. The mRNA is cell-to-cell mobile. | (ZUSAMMEN-CA)-ENHANCED 1 (ZCE1); MLP-LIKE PROTEIN 328 (MLP328) |
| AT2G16700 | Encodes actin depolymerizing factor 5 (ADF5). | ACTIN DEPOLYMERIZING FACTOR 5 (ADF5); (ATADF5) |
| AT1G56510 | TIR-NB-LRR protein that confers resistance to four races of Albugo candida. The mRNA is cell-to-cell mobile. | ACTIVATED DISEASE RESISTANCE 2 (ADR2); WHITE RUST RESISTANCE 4 (WRR4) |
| AT5G04720 | Encodes a member of the ADR1 family nucleotide-binding leucine-rich repeat (NB-LRR) immune receptors. The mRNA is cell-to-cell mobile. | ADR1-LIKE 2 (ADR1-L2); PHOENIX 21 (PHX21) |
| AT2G37760 | Encodes an NADPH-dependent aldo-keto reductase that can act on a wide variety of substrates in vitro including aliphatic and aromatic aldehydes and steroids. Transcript levels for this gene are up-regulated in response to cold, salt, and drought stress. | ALDO-KETO REDUCTASE FAMILY 4 MEMBER C8 (AKR4C8) |
| AT3G22370 | Encodes AOX1a, an isoform of alternative oxidase that is expressed in rosettes, flowers, and root. The alternative oxidase of plant mitochondria transfers electrons from the ubiquinone pool to oxygen without energy conservations. It is regulated through transcriptional control and by pyruvate. Plays a role in shoot acclimation to low temperature. Also is capable of ameliorating reactive oxygen species production when the cytochrome pathway is inhibited. AOX1a also functions as a marker for mitochondrial retrograde response. The mRNA is cell-to-cell mobile. | ALTERNATIVE OXIDASE 1A (AOX1A); (AtHSR3); HYPER-SENSITIVITY-RELATED 3 (HSR3); (ATAOX1A) |
| AT5G27610 | protein ALWAYS EARLY 1 | ALWAYS EARLY 1 (ALY1); ARABIDOPSIS THALIANA ALWAYS EARLY 1 (ATALY1) |
| AT2G38290 | encodes a high-affinity ammonium transporter, which is expressed in shoot and root. Expression in root and shoot is under nitrogen and carbon dioxide regulation, respectively. | AMMONIUM TRANSPORTER 2 (ATAMT2); AMMONIUM TRANSPORTER 2;1 (AMT2;1); AMMONIUM TRANSPORTER 2 (AMT2) |
| AT4G04610 | Encodes a protein disulfide isomerase-like (PDIL) protein, a member of a multigene family within the thioredoxin (TRX) superfamily. This protein also belongs to the adenosine 5'-phosphosulfate reductase-like (APRL) group. The mRNA is cell-to-cell mobile. | APS REDUCTASE 1 (APR1); (APR); PAPS REDUCTASE HOMOLOG 19 (PRH19); (ATAPR1) |
| AT4G21990 | Encodes a protein disulfide isomerase-like (PDIL) protein, a member of a multigene family within the thioredoxin (TRX) superfamily. This protein also belongs to the adenosine 5'-phosphosulfate reductase-like (APRL) group. | APS REDUCTASE 3 (APR3); (PRH-26); (ATAPR3); PAPS REDUCTASE HOMOLOG 26 (PRH26) |
| AT2G05440 | GLYCINE RICH PROTEIN 9 | ARABIDOPSIS GLYCINE RICH PROTEIN 9 (ATGRP9); GLYCINE RICH PROTEIN 9 (GRP9) |
| AT2G40000 | ortholog of sugar beet HS1 PRO-1 2 | ARABIDOPSIS ORTHOLOG OF SUGAR BEET HS1 PRO-1 2 (ATHSPRO2);ORTHOLOG OF SUGAR BEET HS1 PRO-1 2 (HSPRO2) |
| AT1G22500 | Gene encodes a putative C3HC4-type RING zinc finger factor. it is induced in response to light and ascorbate stimulus. | ARABIDOPSIS THALIANA ARABIDOPSIS TOXICOS EN LEVADURA 15 (AtATL15); ARABIDOPSIS TOXICOS EN LEVADURA 15 (ATL15) |
| AT1G22400 | UDP-Glycosyltransferase superfamily protein | ARABIDOPSIS THALIANA UDP-GLUCOSYL TRANSFERASE 85A1 (ATUGT85A1); (UGT85A1) |
| AT5G52830 | Encodes a WRKY transcription factor WRKY27. Mutation in Arabidopsis WRKY27 results in delayed symptom development in response to the bacterial wilt pathogen Ralstonia solanacearum. | ARABIDOPSIS THALIANA WRKY DNA-BINDING PROTEIN 27 (ATWRKY27); WRKY DNA-BINDING PROTEIN 27 (WRKY27) |
| AT4G08870 | Encodes one of the two arginases in the genome. Gene expression is enhanced by methyl jasmonate treatment. It is involved in the defense response to B.cinerea. | ARGININE AMIDOHYDROLASE 2 (ARGAH2) |
| AT3G22890 | encodes ATP sulfurylase, the first enzyme in the sulfate assimilation pathway of Arabidopsis. It may also participate in selenium metabolism. The mRNA is cell-to-cell mobile. | ATP SULFURYLASE 1 (APS1); (ATPS1) |
| AT4G27830 | Encodes a beta-glucosidase that may be responsible for acyl-glucose-dependent anthocyanin glucosyltransferase activity in Arabidopsis. In vitro efforts to demonstrate AAGT activity for BGLU10 have been unsuccessful but experiments with mutants in this gene suggest at least an indirect involvement in anthocyanin formation. | BETA GLUCOSIDASE 10 (BGLU10); (ATBGLU10) |
| AT1G02850 | beta glucosidase 11 | BETA GLUCOSIDASE 11 (BGLU11) |
| AT1G52400 | encodes a member of glycosyl hydrolase family 1, located in inducible ER bodies which were formed after wounding, required in inducible ER body formation the mRNA is cell-to-cell mobile. | BETA-GLUCOSIDASE HOMOLOG 1 (BGL1); A. THALIANA BETA-GLUCOSIDASE 1 (ATBG1); BETA GLUCOSIDASE 18 (BGLU18) |
| AT3G61190 | Encodes a protein with a C2 domain that binds to BON1 in yeast two hybrid analyses. Its ability to bind to phospholipids is enhanced by calcium ions. Involved in maintaining cell homeostasis. | BON ASSOCIATION PROTEIN 1 (BAP1) |
| AT5G49480 | AtCP1 encodes a novel Ca2+-binding protein, which shares sequence similarities with calmodulins. The expression of AtCP1 is induced by NaCl. The mRNA is cell-to-cell mobile. | CA2+-BINDING PROTEIN 1 (CP1); CA2+-BINDING PROTEIN 1 (ATCP1) |
| AT2G41010 | Encodes a novel calmodulin binding protein whose gene expression is induced by dehydration and ionic (salt) and non-ionic (mannitol) osmotic stress. Lines over-expressing this gene are more sensitive and anti-sense lines are more tolerant to osmotic stress, suggesting this gene may be a negative regulator of response to osmotic stress. | CALMODULIN (CAM)-BINDING PROTEIN OF 25 KDA (CAMBP25); CALMODULIN (CAM)-BINDING PROTEIN OF 25 KDA (ATCAMBP25) |
| AT5G16080 | carboxyesterase 17 | CARBOXYESTERASE 17 (CXE17); CARBOXYESTERASE 17 (AtCXE17) |
| AT4G15610 | Uncharacterized protein family (UPF0497) | CASP-LIKE PROTEIN 1D1 (CASPL1D1) |
| AT1G55850 | encodes a protein similar to cellulose synthase the mRNA is cell-to-cell mobile. | CELLULOSE SYNTHASE LIKE E1 (CSLE1); (ATCSLE1) |
| AT2G43570 | chitinase | CHITINASE, PUTATIVE (CHI) |
| AT1G72680 | cinnamyl-alcohol dehydrogenase | CINNAMYL ALCOHOL DEHYDROGENASE 1 (ATCAD1); CINNAMYL-ALCOHOL DEHYDROGENASE (CAD1) |
| AT3G62410 | CP12-2 encodes a small peptide found in the chloroplast stroma. It belongs to the CP12 gene family thought to be involved in the formation of a supramolecular complex with glyceraldehyde-3-phosphate dehydrogenase (GAPDH) and phosphoribulokinase (PRK) embedded in the Calvin cycle. CP12-2 is coordinately regulated by light with the photosynthetic GAPDH and PRK. The annotation of this gene is based on article 32494. The mRNA is cell-to-cell mobile. | CP12 DOMAIN-CONTAINING PROTEIN 1 (CP12); CP12 DOMAIN-CONTAINING PROTEIN 2 (CP12-2) |
| AT1G66160 | CYS, MET, PRO, and GLY protein 1 | CYS, MET, PRO, AND GLY PROTEIN 1 (CMPG1); (ATCMPG1) |
| AT4G23260 | Encodes a cysteine-rich receptor-like protein kinase. | CYSTEINE-RICH RLK (RECEPTOR-LIKE PROTEIN KINASE) 18 (CRK18) |
| AT1G56060 | CYSTM3 is a mitochondrial protein that is induced by salt stress and is a negative regulator of salt stress. | CYSTEINE-RICH TRANSMEMBRANE MODULE 3 (ATHCYSTM3) |
| AT2G32190 | cysteine-rich/transmembrane domain A-like protein | CYSTEINE-RICH TRANSMEMBRANE MODULE 4 (ATHCYSTM4) |
| AT3G14680 | putative cytochrome P450 | CYTOCHROME P450, FAMILY 72, SUBFAMILY A, POLYPEPTIDE 14 (CYP72A14) |
| AT3G28740 | Encodes a member of the cytochrome p450 family. Expression is upregulated in response to cis-jasmonate treatment. Overexpression induces synthesis of volatile compounds that affect chemical ecology and insect interactions. | CYTOCHROME P450, FAMILY 81, SUBFAMILY D, POLYPEPTIDE 11 (CYP81D11) |
| AT4G37370 | member of CYP81D | CYTOCHROME P450, FAMILY 81, SUBFAMILY D, POLYPEPTIDE 8 (CYP81D8) |
| AT3G03470 | member of CYP89A | CYTOCHROME P450, FAMILY 87, SUBFAMILY A, POLYPEPTIDE 9 (CYP89A9) |
| AT3G61630 | CRF6 encodes one of the six cytokinin response factors. CRF5 belongs to the AP2/ERF superfamily of the transcriptional factors. CRF proteins rapidly relocalize to the nucleus in response to cytokinin. Analysis of loos-of-function mutants revealed that the CRFs function redundantly to regulate the development of embryos, cotyledons and leaves. | CYTOKININ RESPONSE FACTOR 6 (CRF6) |
| AT3G49620 | encodes a protein similar to 2-oxoacid-dependent dioxygenase. Expression is induced after 24 hours of dark treatment, in senescing leaves and treatment with exogenous photosynthesis inhibitor. Induction of gene expression was suppressed in excised leaves supplied with sugar. The authors suggest that the gene's expression pattern is responding to the level of sugar in the cell. | DARK INDUCIBLE 11 (DIN11) |
| AT3G60140 | Encodes a protein similar to beta-glucosidase and is a member of glycoside hydrolase family 1. Expression is induced after 24 hours of dark treatment, in senescing leaves and treatment with exogenous photosynthesis inhibitor. Induction of gene expression was suppressed in excised leaves supplied with sugar. The authors suggest that the gene's expression pattern is responding to the level of sugar in the cell. The mRNA is cell-to-cell mobile. | DARK INDUCIBLE 2 (DIN2); SENESCENCE-RELATED GENE 2 (SRG2); BETA GLUCOSIDASE 30 (BGLU30) |
| AT5G45380 | urea-proton symporter DEGRADATION OF UREA 3 (DUR3) | DEGRADATION OF UREA 3 (DUR3); (ATDUR3) |
| AT1G21910 | encodes a member of the DREB subfamily A-5 of ERF/AP2 transcription factor family. The protein contains one AP2 domain. There are 15 members in this subfamily including RAP2.1, RAP2.9 and RAP2.10. | DEHYDRATION RESPONSE ELEMENT-BINDING PROTEIN 26 (DREB26) |
| AT1G48320 | Encodes one of the two functional DHNA-CoA (1,4-dihydroxy-2-naphthoyl-CoA) thioesterases found in Arabidopsis. | DHNA-COA THIOESTERASE 1 (DHNAT1) |
| AT2G36800 | Encodes a DON-Glucosyltransferase. The UGT73C5 glucosylates both brassinolide and castasterone in the 23-O position. The enzyme is presumably involved in the homeostasis of those steroid hormones hence regulating BR activity. Transgenic plants overexpressing UGT73C5 show a typical BR-deficient phenotype. | DON-GLUCOSYLTRANSFERASE 1 (DOGT1); UDP-GLUCOSYL TRANSFERASE 73C5 (UGT73C5) |
| AT2G23340 | encodes a member of the DREB subfamily A-5 of ERF/AP2 transcription factor family. The protein contains one AP2 domain. There are 16 members in this subfamily including RAP2.1, RAP2.9 and RAP2.10. | DREB AND EAR MOTIF PROTEIN 3 (DEAR3) |
| AT4G12480 | Encodes a putative lipid transfer protein, vernalization-responsive and cold-induced. It is involved in priming the SAR and ISR responses, specifically in propagating the cell-to-cell mobile signal. | EARLY ARABIDOPSIS ALUMINUM INDUCED 1 (EARLI1); (pEARLI 1) |
| AT2G40080 | Encodes a novel nuclear 111 amino-acid phytochrome-regulated component of a negative feedback loop involving the circadian clock central oscillator components CCA1 and LHY. ELF4 is necessary for light-induced expression of both CCA1 and LHY, and conversely, CCA1 and LHY act negatively on light-induced ELF4 expression. ELF4 promotes clock accuracy and is required for sustained rhythms in the absence of daily light/dark cycles. It is involved in the phyB-mediated constant red light induced seedling de-etiolation process and may function to coregulate the expression of a subset of phyB-regulated genes. | EARLY FLOWERING 4 (ELF4) |
| AT1G08930 | encodes a putative sucrose transporter whose gene expression is induced by dehydration and cold. The mRNA is cell-to-cell mobile. | EARLY RESPONSE TO DEHYDRATION 6 (ERD6) |
| AT1G07000 | A member of EXO70 gene family, putative exocyst subunits, conserved in land plants. Arabidopsis thaliana contains 23 putative EXO70 genes, which can be classified into eight clusters on the phylogenetic tree. | EXOCYST SUBUNIT EXO70 FAMILY PROTEIN B2 (EXO70B2); EXOCYST SUBUNIT EXO70 FAMILY PROTEIN B2 (ATEXO70B2) |
| AT5G61010 | A member of EXO70 gene family, putative exocyst subunits, conserved in land plants. Arabidopsis thaliana contains 23 putative EXO70 genes, which can be classified into eight clusters on the phylogenetic tree. | EXOCYST SUBUNIT EXO70 FAMILY PROTEIN E2 (EXO70E2); EXOCYST SUBUNIT EXO70 FAMILY PROTEIN E2 (ATEXO70E2) |
| AT1G26380 | Functions in the biosynthesis of 4-hydroxy indole-3-carbonyl nitrile (4-OH-ICN), a cyanogenic phytoalexin in Arabidopsis. FOX1 acts as a dehydrogenase on indole cyanohydrin to form indole carbonyl nitrile. | FAD-LINKED OXIDOREDUCTASE (FOX); (ATBBE3); FAD-LINKED OXIDOREDUCTASE 1 (FOX1) |
| AT4G30530 | Encodes a gamma-glutamyl peptidase, outside the GGT family, that can hydrolyze gamma-glutamyl peptide bonds. The mRNA is cell-to-cell mobile. | GAMMA-GLUTAMYL PEPTIDASE 1 (GGP1) |
| AT1G74360 | NILR1 encodes a serine/threonine kinase involved in defense response to nematodes. | GERMINATION REPRESSION AND CELL EXPANSION RECEPTOR-LIKE KINASE (GRACE); NEMATODE-INDUCED LRR-RLK 1 (NILR1) |
| AT1G69930 | Encodes glutathione transferase belonging to the tau class of GSTs. Naming convention according to Wagner et al. (2002). | GLUTATHIONE S-TRANSFERASE TAU 11 (GSTU11); GLUTATHIONE S-TRANSFERASE TAU 11 (ATGSTU11) |
| AT1G17170 | Encodes glutathione transferase belonging to the tau class of GSTs. Naming convention according to Wagner et al. (2002). It is involved in the detoxification of the environmental pollutant 2,4,6-trinitrotoluene. Arabidopsis plants over-expressing At1g17170 were more resistant to TNT, removed more TNT from sterile and soil-based media, and had reduced levels of glutathione when grown in the presence of TNT. | GLUTATHIONE S-TRANSFERASE TAU 24 (ATGSTU24); GLUTATHIONE S-TRANSFERASE TAU 24 (GSTU24); ARABIDOPSIS THALIANA GLUTATHIONE S-TRANSFERASE (CLASS TAU) 24 (GST) |
| AT2G29420 | Encodes glutathione transferase belonging to the tau class of GSTs. Naming convention according to Wagner et al. (2002). Induced by Salicylic acid. Independent of NPR1 for their induction by salicylic acid. | GLUTATHIONE S-TRANSFERASE TAU 7 (ATGSTU7); GLUTATHIONE S-TRANSFERASE TAU 7 (GSTU7); GLUTATHIONE S-TRANSFERASE 25 (GST25) |
| AT5G13370 | IBA - specific acyl acid amido synthetase which conjugates glutamine to IBA. It is involved in generating inactive and/or storage forms of IBA in the seedling, root, and silique. May play a role in auxin homeostasis by modulating the levels of IBA for peroxisomal conversion to IAA. | GRETCHEN HAGEN 3.15 (GH3.15) |
| AT1G69720 | Encodes a member (HO3) of the heme oxygenase family. | HEME OXYGENASE 3 (ho3) |
| AT2G17820 | Encodes a member of the histidine kinase family. | HISTIDINE KINASE 1 (HK1); (AHK1); HISTIDINE KINASE 1 (ATHK1) |
| AT2G04400 | Acts during tryptophan biosynthesis controlled by ERF109. | INDOLE-3-GLYCEROL PHOSPHATE SYNTHASE (IGPS) |
| AT4G18010 | Encodes an inositol polyphosphate 5-phosphatase that appears to have Type I activity. It can dephosphorylate IP3(inositol (1,4,5) P3) and IP4 (inositol (1,3,4,5) P4), but it does not appear to be active against phosphatidylinositol 4,5 bisphosphate. Overexpression of this gene renders plants insensitive to ABA in germination and growth assays. | INOSITOL (1,4,5) P3 5-PHOSPHATASE II (IP5PII); MYO-INOSITOL POLYPHOSPHATE 5-PHOSPHATASE 2 (5PTASE2); MYO-INOSITOL POLYPHOSPHATE 5-PHOSPHATASE 2 (AT5PTASE2) |
| AT5G13220 | Plants overexpressing At5g13220.3, but not At5g13220.1 showed enhanced insensitivity to MeJa. | JASMONATE-ASSOCIATED 1 (JAS1); JASMONATE-ZIM-DOMAIN PROTEIN 10 (JAZ10); TIFY DOMAIN PROTEIN 9 (TIFY9) |
| AT4G02410 | Concanavalin A-like lectin protein kinase family protein | LECTIN-LIKE PROTEIN KINASE 1 (LPK1); L-TYPE LECTIN RECEPTOR KINASE IV.3 (LECRK-IV.3); (ATLPK1) |
| AT4G34950 | Major facilitator superfamily protein | MAJOR FACILITATOR SUPERFAMILY 1 (MFS1) |
| AT1G73500 | member of MAP Kinase Kinase family. Auto phosphorylates and also phosphorylates MPK3 and MPK6. Independently involved in ethylene and calmalexin biosynthesis. Induces transcription of ACS2, ACS6, ERF1, ERF2, ERF5, ERF6, CYP79B2, CYP79B3, CYP71A13 and PAD3. | MAP KINASE KINASE 9 (MKK9); (ATMKK9) |
| AT3G09390 | metallothionein, binds to and detoxifies excess copper and other metals, limiting oxidative damage | METALLOTHIONEIN 2A (MT2A); ARABIDOPSIS THALIANA METALLOTHIONEIN-1 (ATMT-1); ARABIDOPSIS THALIANA METALLOTHIONEIN-K (ATMT-K) |
| AT3G23250 | Member of the R2R3 factor gene family. | MYB DOMAIN PROTEIN 15 (MYB15); MYB DOMAIN PROTEIN 15 (ATMYB15); (ATY19) |
| AT2G39030 | Encodes a protein that acts as an ornithine N-delta-acetyltransferase, leading to the formation of N-delta-actetylornithine. This compound is likely used in plant defense and levels of it are increased in Arabidopsis plants in response to MeJA and ABA. The mRNA is cell-to-cell mobile. | N-ACETYLTRANSFERASE ACTIVITY 1 (NATA1) |
| AT5G39610 | Encodes a NAC-domain transcription factor. Positively regulates aging-induced cell death and senescence in leaves. This gene is upregulated in response to salt stress in wildtype as well as NTHK1 transgenic lines although in the latter case the induction was drastically reduced. It was also upregulated by ABA, ACC and NAA treatment, although in the latter two cases, the induction occurred relatively late when compared with NaCl or ABA treatments. Note: this protein (AtNAC6) on occasion has also been referred to as AtNAC2, not to be confused with the AtNAC2 found at locus AT3G15510. | NAC DOMAIN CONTAINING PROTEIN 2 (ATNAC2); ORESARA 1 (ORE1); NAC DOMAIN CONTAINING PROTEIN 6 (NAC6); NAC DOMAIN CONTAINING PROTEIN 6 (ATNAC6); NAC DOMAIN CONTAINING PROTEIN 2 (NAC2); ARABIDOPSIS NAC DOMAIN CONTAINING PROTEIN 92 (ANAC092) |
| AT5G63790 | Encodes a member of the NAC family of transcription factors. ANAC102 appears to have a role in mediating response to low oxygen stress (hypoxia) in germinating seedlings. Its expression can be induced by beta-cyclocitral, an oxidized by-product of beta-carotene generated in the chloroplasts, mediates a protective retrograde response that lowers the levels of toxic peroxides and carbonyls, limiting damage to intracellular components. | NAC DOMAIN CONTAINING PROTEIN 102 (NAC102); NAC DOMAIN CONTAINING PROTEIN 102 (ANAC102) |
| AT1G52890 | encodes a NAC transcription factor whose expression is induced by drought, high salt, and abscisic acid. This gene binds to ERD1 promoter in vitro. | NAC DOMAIN CONTAINING PROTEIN 19 (NAC019); (ANAC19); NAC DOMAIN CONTAINING PROTEIN 19 (ANAC019) |
| AT5G13180 | Encodes a NAC domain transcription factor that interacts with VND7 and negatively regulates xylem vessel formation. | NAC DOMAIN CONTAINING PROTEIN 83 (ANAC083); NAC DOMAIN CONTAINING PROTEIN 83 (NAC083); VND-INTERACTING 2 (VNI2) |
| AT1G28380 | This gene is predicted to encode a protein involved in negatively regulating salicylic acid-related defense responses and cell death programs. nsl1 mutants develop necrotic lesions spontaneously and show other features of a defense response, such as higher levels of SA and disease resistance-related transcripts, in the absence of a biotic stimulus. The NSL1 protein is predicted to have a MACPF domain, found in proteins that form a transmembrane pore in mammalian immune responses. NSL1 transcript levels do not appear to change in response to biotic stresses, but are elevated by cycloheximide in seedlings, and by sodium chloride in roots. The mRNA is cell-to-cell mobile. | NECROTIC SPOTTED LESIONS 1 (NSL1) |
| AT4G21680 | Encodes a nitrate transporter (NRT1.8). Functions in nitrate removal from the xylem sap. Mediates cadmium tolerance. | NITRATE TRANSPORTER 1.8 (NRT1.8); (ATNPF7.2); NRT1/ PTR FAMILY 7.2 (NPF7.2) |
| AT1G68570 | NPF3.1 is a membrane localized GA transporter that is expressed in the root endodermis. | NRT1/ PTR FAMILY 3.1 (NPF3.1); (ATNPF3.1) |
| AT3G54950 | Encodes pPLAIIIbeta, a member of the Group 3 patatin-related phospholipases. pPLAIIIbeta hydrolyzes phospholipids and galactolipids and additionally has acyl-CoA thioesterase activity. Alterations of pPLAIII result in changes in lipid levels and composition. | PATATIN-LIKE PROTEIN 7 (PLP7); PATATIN-RELATED PHOSPHOLIPASE IIIBETA (pPLAIIIbeta); PATATIN-LIKE PROTEIN 6 (PLA IIIA) |
| AT1G35140 | EXL1 is involved in the C-starvation response. Phenotypic changes of an exl1 loss of function mutant became evident only under corresponding experimental conditions. For example, the mutant showed diminished biomass production in a short-day/low light growth regime, impaired survival during extended night, and impaired survival of anoxia stress. | PHOSPHATE-INDUCED 1 (PHI-1); EXORDIUM LIKE 7 (EXL7); EXORDIUM LIKE 1 (EXL1) |
| AT5G29000 | MYB-CC family member. PHL1 acts redundantly with PHR1 to regulate responses to Pi starvation. | PHR1-LIKE 1 (PHL1) |
| AT2G22860 | Phytosulfokine 2 precursor, coding for a unique plant peptide growth factor. The mRNA is cell-to-cell mobile. | PHYTOSULFOKINE 2 PRECURSOR (ATPSK2); PHYTOSULFOKINE 2 PRECURSOR (PSK2) |
| AT5G20240 | Floral homeotic gene encoding a MADS domain transcription factor. Required for the specification of petal and stamen identities. | PISTILLATA (PI) |
| AT4G23190 | Encodes putative receptor-like protein kinase that is induced by the soil-borne vascular bacteria, Ralstonia solanacearum. Naming convention from Chen et al 2003 (PMID 14756307) | RECEPTOR LIKE PROTEIN KINASE 3 (AT-RLK3); CYSTEINE-RICH RLK (RECEPTOR-LIKE PROTEIN KINASE) 11 (CRK11) |
| AT1G09970 | RLK7 belongs to a leucine-rich repeat class of receptor-likekinase (LRR-RLKs). It is involved in the control of germination speed and the tolerance to oxidant stress. The mRNA is cell-to-cell mobile. | RECEPTOR-LIKE KINASE 7 (RLK7); (LRR XI-23) |
| AT1G43160 | encodes a member of the ERF (ethylene response factor) subfamily B-4 of ERF/AP2 transcription factor family (RAP2.6). The protein contains one AP2 domain. There are 7 members in this subfamily. | RELATED TO AP2 6 (RAP2.6) |
| AT5G13330 | encodes a member of the ERF (ethylene response factor) subfamily B-4 of ERF/AP2 transcription factor family. The protein contains one AP2 domain. There are 7 members in this subfamily. | RELATED TO AP2 6L (Rap2.6L) |
| AT2G33380 | Encodes a calcium binding protein whose mRNA is induced upon treatment with NaCl, ABA and in response to desiccation. mRNA expression under drought conditions is apparent particularly in leaves and flowers. Isoform of caleosin with a role as a peroxygenase involved in oxylipin metabolism during biotic and abiotic stress. Involved in the production of 2-hydroxy-octadecatrienoic acid. The peroxygenase has a narrow substrate specificity thus acting as a fatty acid hydroperoxide reductase in vivo. | RESPONSIVE TO DESICCATION 20 (RD20); CALEOSIN 3 (CLO-3); PEROXYGENASE 3 (PXG3); ARABIDOPSIS THALIANA CALEOSIN 3 (AtCLO3); (ATRD20); CALEOSIN 3 (CLO3) |
| AT5G59820 | Encodes a zinc finger protein involved in high light and cold acclimation. Overexpression of this putative transcription factor increases the expression level of 9 cold-responsive genes and represses the expression level of 15 cold-responsive genes, including CBF genes. Also, lines overexpressing this gene exhibits a small but reproducible increase in freeze tolerance. Because of the repression of the CBF genes by the overexpression of this gene, the authors speculate that this gene may be involved in negative regulatory circuit of the CBF pathway. The mRNA is cell-to-cell mobile. | RESPONSIVE TO HIGH LIGHT 41 (RHL41); (ATZAT12); (ZAT12) |
| AT4G17230 | Encodes a scarecrow-like protein (SCL13). Member of GRAS gene family. | SCARECROW-LIKE 13 (SCL13) |
| AT1G55920 | Encodes a chloroplast/cytosol localized serine O-acetyltransferase involved in sulfur assimilation and cysteine biosynthesis. Expressed in the vascular system. The mRNA is cell-to-cell mobile. | SERINE ACETYLTRANSFERASE 2;1 (ATSERAT2;1); (ATSAT1); SERINE ACETYLTRANSFERASE 2;1 (SERAT2;1); SERINE ACETYLTRANSFERASE 1 (SAT1); SERINE ACETYLTRANSFERASE 5 (SAT5) |
| AT2G45210 | SAUR-like auxin-responsive protein family | SMALL AUXIN UPREGULATED 36 (SAUR36); SENESCENCE-ASSOCIATED GENE 201 (SAG201) |
| AT2G21220 | SAUR-like auxin-responsive protein family | SMALL AUXIN UPREGULATED RNA 12 (SAUR12) |
| AT1G75580 | SAUR-like auxin-responsive protein family | SMALL AUXIN UPREGULATED RNA 51 (SAUR51) |
| AT3G60690 | SAUR-like auxin-responsive protein family | SMALL AUXIN UPREGULATED RNA 59 (SAUR59) |
| AT1G78290 | encodes a member of SNF1-related protein kinase (SnRK2) family whose activity is activated by ionic (salt) and non-ionic (mannitol) osmotic stress and dehydration. | SNF1-RELATED PROTEIN KINASE 2-8 (SNRK2-8); SNF1-RELATED PROTEIN KINASE 2C (SRK2C); SNF1-RELATED PROTEIN KINASE 2.8 (SNRK2.8) |
| AT5G24160 | squalene monooxygenase 6 | SQUALENE MONOXYGENASE 6 (SQE6) |
| AT3G28210 | Encodes a putative zinc finger protein (PMZ). | STRESS-ASSOCIATED PROTEIN 12 (SAP12); (PMZ) |
| AT5G20830 | Encodes a protein with sucrose synthase activity (SUS1). | SUCROSE SYNTHASE 1 (SUS1); (atsus1); (ASUS1) |
| AT1G20350 | mitochondrial inner membrane translocase | TRANSLOCASE INNER MEMBRANE SUBUNIT 17-1 (TIM17-1) |
| AT5G54810 | A.thaliana tryptophan synthase beta subunit (trpB) | TRYPTOPHAN SYNTHASE BETA-SUBUNIT 1 (TSB1); (ATTSB1); TRYPTOPHAN BIOSYNTHESIS 2 (TRP2); TRYPTOPHAN BIOSYNTHESIS B (TRPB) |
| AT5G64510 | Encodes Tunicamycin Induced 1(TIN1), a plant-speci‑c ER stress-inducible protein. TIN1 mutation affects pollen surface morphology. Transcriptionally induced by treatment with the N-linked glyclsylation inhibitor tunicamycin. | TUNICAMYCIN INDUCED 1 (TIN1) |
| AT2G02810 | Encodes a multitransmembrane hydrophobic protein that functions as transporter of UDP-galactose and UDP-glucose into the Golgi. Localized in the ER. Involved in the unfolded protein response, a mechanism that controls proper protein folding in the ER. | UDP-GALACTOSE TRANSPORTER 1 (UTR1); UDP-GALACTOSE TRANSPORTER 1 (ATUTR1) |
| AT4G23010 | UDP-galactose transporter 2 | UDP-GALACTOSE TRANSPORTER 2 (UTR2) |
| AT1G14360 | UDP-galactose transporter 3 | UDP-GALACTOSE TRANSPORTER 3 (UTR3); (ATUTR3) |
| AT2G15480 | UDP-glucosyl transferase 73B5 | UDP-GLUCOSYL TRANSFERASE 73B5 (UGT73B5) |
| AT2G30140 | Encodes a putative glycosyltransferase. Regulates flowering time via FLOWERING LOCUS C. | UDP-GLUCOSYL TRANSFERASE 87A2 (UGT87A2) |
| AT2G15490 | UDP-glycosyltransferase 73B4 | UDP-GLYCOSYLTRANSFERASE 73B4 (UGT73B4) |
| AT5G43580 | Predicted to encode a PR (pathogenesis-related) peptide that belongs to the PR-6 proteinase inhibitor family. Functions in resistance to necrotrophic fungi and insect herbivory. Six putative PR-6-type protein encoding genes are found in Arabidopsis: At2g38900, At2g38870, At5g43570, At5g43580, At3g50020 and At3g46860. | UNUSUAL SERINE PROTEASE INHIBITOR (UPI) |
| AT2G47270 | Encodes UPBEAT1 (UPB1), a transcription factor with a bHLH domain. Regulates the expression of a set of peroxidases that modulate the balance of reactive oxygen species (ROS) between the zones of cell proliferation and the zone of cell elongation where differentiation begins. Disruption of UPB1 activity alters this ROS balance, leading to a delay in the onset of differentiation. | UPBEAT1 (UPB1) |
| AT5G07100 | Encodes WRKY DNA-binding protein 26 (WRKY26). | WRKY DNA-BINDING PROTEIN 26 (WRKY26) |
| AT3G01970 | member of WRKY Transcription Factor; Group I | WRKY DNA-BINDING PROTEIN 45 (WRKY45); WRKY DNA-BINDING PROTEIN 45 (ATWRKY45) |
| AT4G30280 | Encodes a xyloglucan endotransglucosylase/hydrolase with only only the endotransglucosylase (XET; EC 2.4.1.207) activity towards xyloglucan and non-detectable endohydrolytic (XEH; EC 3.2.1.151) activity. Expressed in the mature or basal regions of both the main and lateral roots, but not in the tip of these roots where cell division occurs. | XYLOGLUCAN ENDOTRANSGLUCOSYLASE/HYDROLASE 18 (XTH18); XYLOGLUCAN ENDOTRANSGLUCOSYLASE/HYDROLASE 18 (ATXTH18) |
| AT4G25810 | xyloglucan endotransglycosylase-related protein (XTR6) | XYLOGLUCAN ENDOTRANSGLUCOSYLASE/HYDROLASE 23 (XTH23); XYLOGLUCAN ENDOTRANSGLYCOSYLASE 6 (XTR6) |
| AT5G57550 | xyloglucan endotransglycosylase-related protein (XTR3) | XYLOGLUCAN ENDOTRANSGLUCOSYLASE/HYDROLASE 25 (XTH25); XYLOGLUCAN ENDOTRANSGLYCOSYLASE 3 (XTR3) |
| AT4G24120 | Member of a small family of oligopeptide transporters similar to the yellow stripe locus of maize (ZmYS1). | YELLOW STRIPE LIKE 1 (ATYSL1); YELLOW STRIPE LIKE 1 (YSL1) |
| AT1G65730 | Arabidopsis thaliana metal-nicotianamine transporter YSL4 | YELLOW STRIPE LIKE 7 (YSL7) |
| AT1G55910 | member of Putative zinc transporter ZIP2 - like family | ZINC TRANSPORTER 11 PRECURSOR (ZIP11) |
| AT1G25400 | transmembrane protein |  |
| AT2G36780 | UDP-Glycosyltransferase superfamily protein |  |
| AT2G28400 | senescence regulator (Protein of unknown function, DUF584) |  |
| AT5G10580 | plant/protein (Protein of unknown function, DUF599) |  |
| AT5G39580 | Peroxidase superfamily protein |  |
| AT3G14280 | LL-diaminopimelate aminotransferase |  |
| AT1G11210 | cotton fiber protein, putative (DUF761) |  |
| AT4G39675 | hypothetical protein |  |
| AT3G51660 | Tautomerase/MIF superfamily protein |  |
| AT1G69890 | actin cross-linking protein (DUF569) |  |
| AT4G38540 | FAD/NAD(P)-binding oxidoreductase family protein |  |
| AT1G35350 | EXS (ERD1/XPR1/SYG1) family protein |  |
| AT5G39520 | hypothetical protein (DUF1997) |  |
| AT1G23120 | Polyketide cyclase/dehydrase and lipid transport superfamily protein |  |
| AT3G59930 | Encodes a defensin-like (DEFL) family protein. |  |
| AT3G04000 | ChlADR is an aldehyde reductase that catalyzes the reduction of the aldehyde carbonyl groups on saturated and alpha,beta-unsaturated aldehydes with more than 5 carbons in vitro. The N-terminal region of this protein directs GFP to the chloroplast where where ChlADR likely helps to maintain the photosynthetic process by detoxifying reactive carbonyls formed during lipid peroxidation. In addition, this enzyme can also reduce cis-3-hexenal, a major plant volatile compound that contributes to green leaf odor, as well as methylglyoxal in vitro. |  |
| AT5G14730 | hypothetical protein |  |
| AT1G19020 | CDP-diacylglycerol-glycerol-3-phosphate 3-phosphatidyltransferase |  |
| AT5G35735 | Auxin-responsive family protein |  |
| AT1G69050 | hypothetical protein |  |
| AT2G37540 | NAD(P)-binding Rossmann-fold superfamily protein |  |
| AT5G49690 | UDP-Glycosyltransferase superfamily protein |  |
| AT2G16595 | Translocon-associated protein (TRAP), alpha subunit |  |
| AT1G16260 | Wall-associated kinase family protein |  |
| AT5G17760 | P-loop containing nucleoside triphosphate hydrolases superfamily protein |  |
| AT2G43530 | Encodes a defensin-like (DEFL) family protein. The mRNA is cell-to-cell mobile. |  |
| AT1G64610 | Transducin/WD40 repeat-like superfamily protein |  |
| AT3G47540 | Chitinase family protein |  |
| AT4G19880 | Glutathione S-transferase family protein |  |
| AT1G15010 | mediator of RNA polymerase II transcription subunit |  |
| AT5G38940 | RmlC-like cupins superfamily protein |  |
| AT1G51270 | vesicle-associated protein 1-4 |  |
| AT5G55180 | O-Glycosyl hydrolases family 17 protein |  |
| AT3G04640 | glycine-rich protein |  |
| AT3G15760 | cytochrome P450 family protein |  |
| AT2G27660 | Cysteine/Histidine-rich C1 domain family protein |  |
| AT5G10695 | methionyl-tRNA synthetase |  |
| AT1G28600 | GDSL-motif esterase/acyltransferase/lipase. Enzyme group with broad substrate specificity that may catalyze acyltransfer or hydrolase reactions with lipid and non-lipid substrates. |  |
| AT3G28220 | TRAF-like family protein |  |
| AT5G58570 | transmembrane protein |  |
| AT3G51440 | Calcium-dependent phosphotriesterase superfamily protein |  |
| AT2G31945 | transmembrane protein |  |
| AT2G41640 | Glycosyltransferase family 61 protein |  |
| AT3G62260 | Protein phosphatase 2C family protein |  |
| AT2G46600 | Calcium-binding EF-hand family protein |  |
| AT1G10140 | Uncharacterized conserved protein UCP031279 |  |
| AT4G37530 | Peroxidase superfamily protein |  |
| AT5G64110 | Peroxidase superfamily protein |  |
| AT4G27300 | S-locus lectin protein kinase family protein |  |
| AT5G51440 | HSP20-like chaperones superfamily protein |  |
| AT4G12290 | Copper amine oxidase family protein |  |
| AT3G51450 | Calcium-dependent phosphotriesterase superfamily protein |  |
| AT1G76600 | poly polymerase |  |
| AT2G25450 | encodes a protein whose sequence is similar to ACC oxidase |  |
| AT2G25735 | hypothetical protein |  |
| AT5G19440 | similar to Eucalyptus gunnii alcohol dehydrogenase of unknown physiological function (GI:1143445), apple tree, PIR: T16995; NOT a cinnamyl-alcohol dehydrogenase |  |
| AT1G76590 | PLATZ transcription factor family protein |  |
| AT5G54170 | Polyketide cyclase/dehydrase and lipid transport superfamily protein |  |
| AT4G15260 | UDP-Glycosyltransferase superfamily protein |  |
| AT1G09500 | similar to Eucalyptus gunnii alcohol dehydrogenase of unknown physiological function (GI:1143445), Vigna unguiculata (gi:1854445), NOT a cinnamyl-alcohol dehydrogenase |  |
| AT1G72900 | Toll-Interleukin-Resistance (TIR) domain-containing protein |  |
